# Recovering probabilities for nucleotide trimming processes for T cell receptor TRA and TRG V-J junctions analyzed with IMGT tools

**DOI:** 10.1186/1471-2105-9-408

**Published:** 2008-10-02

**Authors:** Kevin Bleakley, Marie-Paule Lefranc, Gérard Biau

**Affiliations:** 1 Institut Curie, Centre de Recherche, Paris, F-75248, France; 2 INSERM, U900, Paris, F-75248, France; 3 Centre for Computational Biology, Ecole des Mines de Paris, 35 rue St Honore, Fontainebleau, F-77305, France; 4 IMGT®, the international ImMunoGeneTics information system, Laboratoire d'ImmunoGénétique Moléculaire LIGM, UPR CNRS 1142, Institut de Génétique Humaine IGH, 141 rue de la Cardonille, 34396 Montpellier Cedex 5, France; 5 LSTA & LMPA, Université Pierre et Marie Curie – Paris VI, Boîte 158, 175 rue du Chevaleret, 75013 Paris, France

## Abstract

**Background:**

Nucleotides are trimmed from the ends of variable (V), diversity (D) and joining (J) genes during immunoglobulin (IG) and T cell receptor (TR) rearrangements in B cells and T cells of the immune system. This trimming is followed by addition of nucleotides at random, forming the N regions (N for nucleotides) of the V-J and V-D-J junctions. These processes are crucial for creating diversity in the immune response since the number of trimmed nucleotides and the number of added nucleotides vary in each B or T cell. IMGT^® ^sequence analysis tools, IMGT/V-QUEST and IMGT/JunctionAnalysis, are able to provide detailed and accurate analysis of the final observed junction nucleotide sequences (tool "output"). However, as trimmed nucleotides can potentially be replaced by identical N region nucleotides during the process, the observed "output" represents a *biased *estimate of the "true trimming process."

**Results:**

A probabilistic approach based on an analysis of the standardized tool "output" is proposed to infer the probability distribution of the "true trimmming process" and to provide plausible biological hypotheses explaining this process. We collated a benchmark dataset of TR alpha (TRA) and TR gamma (TRG) V-J rearranged sequences and junctions analysed with IMGT/V-QUEST and IMGT/JunctionAnalysis, the nucleotide sequence analysis tools from IMGT^®^, the international ImMunoGeneTics information system^®^, . The standardized description of the tool output is based on the IMGT-ONTOLOGY axioms and concepts. We propose a simple first-order model that attempts to transform the observed "output" probability distribution into an estimate closer to the "true trimming process" probability distribution. We use this estimate to test the hypothesis that Poisson processes are involved in trimming. This hypothesis was not rejected at standard confidence levels for three of the four trimming processes: TRAV, TRAJ and TRGV.

**Conclusion:**

By using trimming of rearranged TR genes as a benchmark, we show that a probabilistic approach, applied to IMGT^® ^standardized tool "outputs" opens the way to plausible hypotheses on the events involved in the "true trimming process" and eventually to an exact quantification of trimming itself. With increasing high-throughput of standardized immunogenetics data, similar probabilistic approaches will improve understanding of processes so far only characterized by the "output" of standardized tools.

## Background

The diversity of the chains of immunoglobulins (IG) or antibodies and T cell receptors (TR) depends on several mechanisms [1-10]: first, combinatorial diversity, which is a consequence of the number of variable (V), diversity (D) and joining (J) genes in the IG and TR loci [9,10], second, exonuclease trimming of V, D and J nucleotides and third, addition at random of nucleotides at the V-J and V-D-J junction (N region diversity).

These processes together create a huge diversity in V-J and V-D-J junctions as exemplified by the rearranged IG and TR sequences from IMGT/LIGM-DB [11]. In addition, rearranged V-J and V-D-J genes from IG (but not those from TR) are specifically submitted to the mechanism of somatic hypermutations [9] (IMGT Education, Tutorials, ). The number of different antigen receptors (IG and TR) per individual is estimated to be 2 × 10^12 ^in humans and the only limiting factor seems to be the number of B cells (for the IG) and T cells (for the TR) which is genetically programmed in a given species.

Trimming by exonuclease occurs at the ends of the 3'V-REGION and 5'J-REGION [12] (IMGT labels from the DESCRIPTION axiom of IMGT-ONTOLOGY are in capital letters [13,14]) and at both ends of the D-REGION, present in the IG heavy (IGH), TR beta (TRB) and TR delta (TRD) loci [9,10]. Little is known about the mechanisms that regulate trimming of V, D and J genes during V-J and V-D-J rearrangement. Given the importance of trimming in the creation of the vast diversity of V-J and V-D-J junctions, it is of great interest to better understand this process.

Based on the IMGT-ONTOLOGY axioms and concepts of classification (IMGT gene names) [9,10,15,16], description (IMGT labels) [17,18] and numerotation (IMGT concepts for numbering, in particular, IMGT unique numbering for V, C and G domains) [19-21], on-line tools have been developed by IMGT^®^, the international ImMunoGeneTics information system^®^, [22], for the standardized analysis of immunogenetics data.

**Figure 1:**
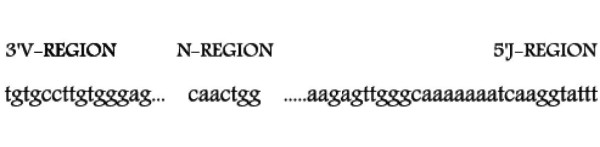
**IMGT^® ^junction analysis ''output'' from IMGT/V-QUEST+JCTA**. A TRA or TRG ''output'' showing the observed post-trimming 3'V-REGION, N region and post-trimming 3'V-REGION. The dots indicate nucleotides trimmed from the 3'V-REGION and 5'J-REGION by comparison with the closest germline V and J genes and alleles identified by IMGT/V-QUEST [23,24] and analysed by IMGT/JunctionAnalysis [25].

Among them, IMGT/V-QUEST is the highly customized and integrated IMGT system for the standardized analysis of rearranged IG and TR sequences [23,24]. IMGT/V-QUEST identifies the V, D and J genes in rearranged V-J and V-D-J sequences. IMGT/V-QUEST integrates IMGT/JunctionAnalysis [25] (noted IMGT/V-QUEST+JCTA hereafter) to provide a detailed analysis of the observed V-J and V-D-J junctions. As bioinformatics tools become higher-throughput (IMGT/V-QUEST+JCTA can process batches of 50 sequences at present and proposes a "Synthesis view" of the results [24]), data representing variables such as *number of trimmed nucleotides *and *N-REGION length *(number of added nucleotides) can be obtained [12]. However, these numbers represent what is observed in the final "output" but do not necessarily represent the extent of the "true" trimming or nucleotide addition processes. Indeed, randomly trimmed nucleotides can be replaced by *identical *randomly added N region nucleotides. As a consequence, the number of trimmed V or J nucleotides (represented by the dots in Figure 1) will sometimes be underestimated.

There is therefore a need to quantify this bias if we want to investigate the underlying processes. The goal of the present article is to explore this possibility using TRA and TRG trimming processes, where only V and J genes are involved [10].

Our strategy is the following: given an IMGT/V-QUEST+JCTA standardized output, we aim to calculate the probabilities of all possible trimming events that are consistent with this output. Then, using many such outputs, we aim to probabilistically transform the set of tool "output" data into a representation of the "true trimming process" (i.e., the amount of trimming that *actually *occurred). This probabilistic framework appears naturally by first taking the "output" dataset and simply calculating the empirical probability that the tool "output" shows that 0,1,2... nucleotides were trimmed. Then, understanding how the tool works, we aim to "correct" these empirical probabilities with respect to the tool's biases. A comprehensive introduction to probability distributions (empirical, true) can be found in [26,27] and a simple introduction to Bernoulli and Poisson distributions is included in Supplementary Data [see Additional file 1].

A first-order model is presented in Results, along with statistical tests on the transformed probability distributions. A proof of the first-order model and a proposed second-order model (also with proof) can be found in Supplementary Data [see Additional file 1].

## Results and discussion

### A first-order model

**Figure 2:**
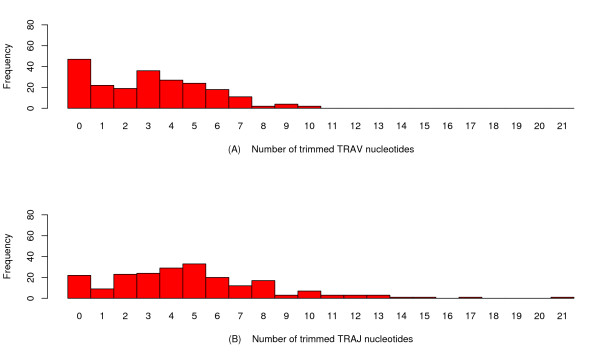
**TRA trimming distribution for the IMGT/V-QUEST+JCTA output datasets**. Histograms of the number of trimmed V nucleotides and number of trimmed J nucleotides for the set of 212 human rearranged TRAV-TRAJ junction sequences.

**Figure 3:**
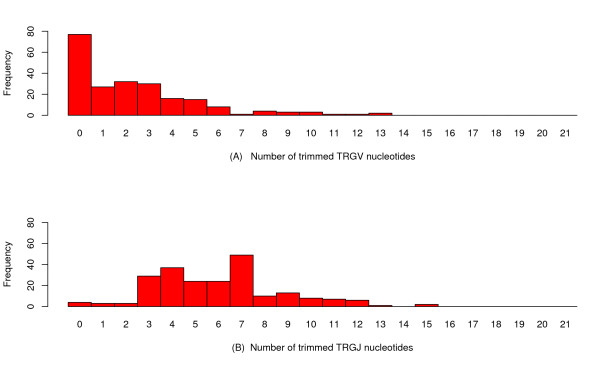
**TRG trimming distribution for the IMGT/V-QUEST+JCTA output datasets**. Histograms of the number of trimmed V nucleotides and number of trimmed J nucleotides for the set of 220 human rearranged TRGV-TRGJ junction sequences.

Figures 2 and 3 show histograms of the number of trimmed TRAV, TRAJ, TRGV and TRGJ nucleotides obtained from 212 TRAV-TRAJ and 220 TRGV-TRGJ junction sequences analysed by IMGT/V-QUEST+JCTA and whose results were agreed upon by experts.

As potentially more nucleotides are trimmed in the "true process" than appear to have been trimmed according to the tool "output," we would like to transform the "output" data into "true process" data.

A factor also to take into consideration are the quantities of data at zero (except for TRGJ), which do not match the relatively smooth form of the tool "output" data distributions (see Figures 2 and 3). This may be evidence of a two-step process: either the trimming process is activated, or not. If activated, it follows some as yet unknown law. If not, no trimming occurs. Obviously, if the unknown law also takes the value zero, the fraction of data that takes the value zero would then have two sources (either the first process is not activated, or is activated and the second process gives the value zero). Thankfully, as will be shown under the following first-order model, probabilistically transforming the "output" distribution towards the "true process" distribution (under the hypotheses of the model) does not cause further complications. Indeed, the transformed masses (i.e., fractions of the total number of data found at each possible data value) *above zero *do not depend on the original fraction *at zero*. This means that performing maximum likelihood estimation of the parameters of a two-step process is well-defined on the transformed data.

#### Recovering an estimation of the true process probability distribution

Here we introduce a mathematical result that allows us to recover an estimation of the true process probability distribution of the number of trimmed V nucleotides. This result is almost (but not entirely) valid for the true process probability distribution of the number of trimmed J nucleotides. The potential problem is that IMGT/V-QUEST+JCTA selects the J gene after the V gene (see Methods and [25] for more details), thus there is a non-zero chance that 5'J-REGION nucleotides will accidentally be included in the V gene prediction when there has been no N region nucleotide addition. After reanalyzing the data, we found that in the TRAV-TRAJ dataset, this happened at most 3 times and thus was rare enough to be ignored. However, for the TRGV-TRGJ data, this potentially happened quite often, so estimated probability distribution results for the TRGJ trimming process must be used with caution.

Let ℙ {*B *= *k*} mean 'the probability that *k *3'V-REGION nucleotides are trimmed under the (unknown) true trimming process distribution *f*_*B*_'. We want to estimate this for *k *≥ 0. Let ℙ {*F *= *i*} mean 'the probability that *i *nucleotides appear *k *to have been trimmed.' That is, the random variable *F *represents the 3'V-REGION trimming distribution of the tool "output." We do not know the distribution *f*_*F *_of *F *exactly, but through our datasets we have an empirical estimate of it.

The goal is to use this empirical estimate of *f*_*F *_to estimate *f*_*B*_. To begin, Theorem 1 [see Additional file 1] shows that under some simple hypotheses (the 'first-order' model), there is an explicit link between the law of the observed 3'V-REGION tool "output" trimming distribution and the "true" (or more correctly, "bias-corrected": technically, it is "true" only if the hypotheses of the first-order model hold in general) process distribution. Indeed, for any *k *≥ 1 we find:

$$\mathbb{P}\{B=k\}=\frac{4}{3}\mathbb{P}\{F=k\}-\frac{1}{3}\mathbb{P}\{F=k+1\},$$

and for *k *= 0 we find:

$$\mathbb{P}\{B=0\}=\mathbb{P}\{F=0\}-\frac{1}{3}\mathbb{P}\{F=1\}.$$

We call this the (4/3, 1/3) rule. Supposing the first-order hypotheses are correct, we would have for example that the bias-corrected probability that 5 V nucleotides were trimmed is equal to (4/3) the probability the tool "output" gives 5 trimmed nucleotides minus (1/3) the probability it gives 6 trimmed nucleotides. We see indeed that under these hypotheses, transformed fractions of data at each data value *above zero *do not depend on the original fraction of data *at zero*.

We remark that it is unlikely that the probabilities of appearance of A, C, G and T nucleotides in the N region are equal (= 1/4, as is assumed in the first-order model), nor in the 3'V-REGION or 5'J-REGION. A second-order model, giving much more freedom to possible A, C, G and T frequencies (each frequency taking some value between 1/6 and 1/3) can be found in Supplementary Data [see Additional file 1]. In brief, we find that the first-order model approximates well the more general second-order model. Thus for simplicity, the first-order result can be used in the place of the second-order result to form hypotheses on trimming processes.

### Testing the transformed V and J trimming distributions

Under the hypotheses of the first-order model, we transformed the TRA and TRG tool "output" data following the law *f*_*F *_into probability distributions following the law *f*_*B*_.

Remarking that apart from at zero, these transformed results often resembled Poisson laws, we attempted to formally test this. More precisely, we supposed that we were dealing with a Bernoulli process (with parameter *p *unknown) followed by a Poisson process (parameter *λ *unknown) if the Bernoulli process gave a success. This meant a density function of:

$$\begin{array}{cc} f(x,p,\lambda )=(1-p)1_{\left\{x=0\right\}}+p\frac{e^{-\lambda }\lambda^{x}}{x!}, & x=0,1,2,... \end{array}$$

Maximum likelihood was then performed in order to simultaneously estimate the parameters *p *and *λ*, this being necessary to subsequently test the hypothesis that we are dealing with a two-step Bernoulli-Poisson process having parameters *p *and *λ*.

Given data *x*_1_, *x*_2_,..., *x*_*n*_, it is easy to show that maximum likelihood estimation gives the equations *g*(*λ*) = (1 - exp(-*λ*))*C *- *mλ *= 0 and *p *= *m*/*n*(1 - exp(-*λ*)) to be solved, where *m *is the number of *x*_*i *_> 0 and *C *the sum of the values of the *x*_*i *_> 0. As *m *and *C *are thus constants given any dataset, we see that resolving *g*(*λ*) = 0 for *λ *then allows us to solve for *p *in the second equation. Upon performing the first-order transformation, we found (*m*, *C*) = (517/3, 708), (580/3, 3286/3), (152, 1682/3), (670/3, 4238/3) for the TRAV, TRAJ, TRGV and TRGJ datasets, respectively.

To see that *g*(*λ*) = 0 has a unique solution (and thus *p *also) here, we first remark that for each of these *m*, *C *> 0, lim_*λ*→0 _*g'*(*λ*) > 0 and *g''*(*λ*) < 0 for *λ *> 0, lim_*λ*→∞_*g'*(*λ*) = -*m *< 0, and *g'*(*λ*) is a continuous function for *λ *> 0. Thus, by the intermediate value theorem, there exists at least one *λ *> 0 such that *g'*(*λ*) = 0, and since *g''*(*λ*) < 0 for *λ *> 0, there is in fact a unique solution, which can be easily found numerically for each given *m*, *C *> 0. Indeed, we find (*p*, *λ*) = (0.83, 4.04), (0.92, 5.65), (0.71, 3.59), (1, 6.31) for the TRAV, TRAJ, TRGV and TRGJ datasets, respectively.

**Figure 4:**
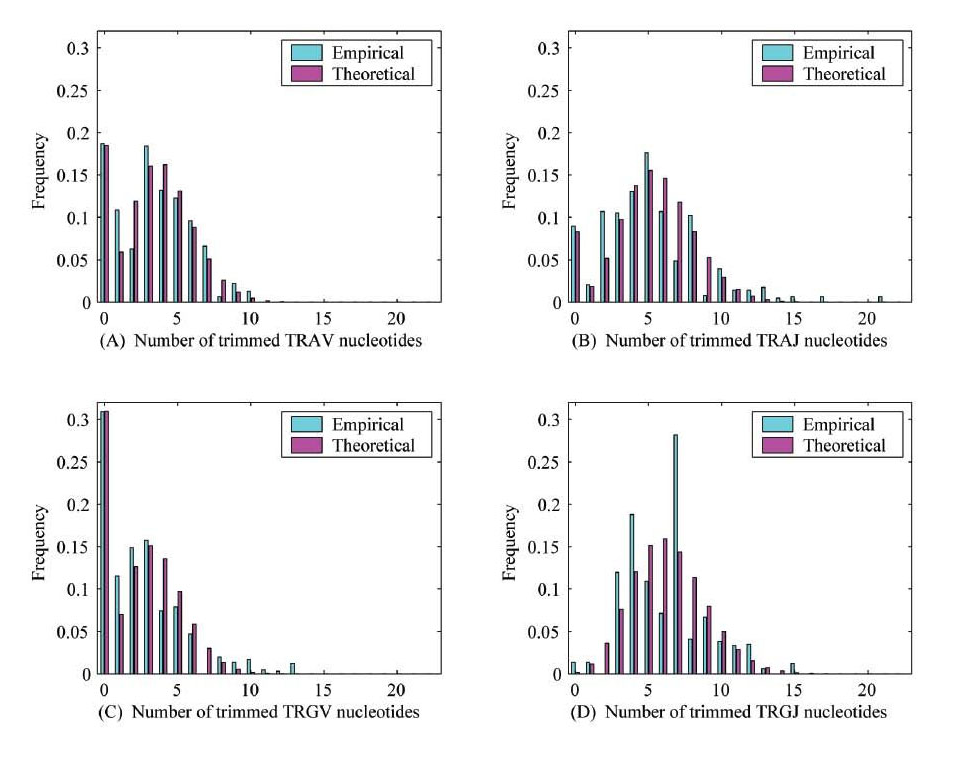
**Comparing "bias-corrected" distributions with Poisson distributions**. First-order "bias-corrected" distributions for TRAV, TRAJ, TRGV and TRGJ compared with theoretical Poisson distributions.

Figure 4 shows the transformed distributions (blue) and the corresponding theoretical predictions (pink) for the Bernoulli-Poisson distribution *f *in each of the four cases. We tested the four empirical distributions against the theoretical Bernoulli-Poisson distribution *f *using Pearson's χ^2 ^test. The null hypothesis ${ℋ}_{0}$ is that the distribution follows *f *with parameters (*p*, *λ*). In order to keep within the assumptions of the test, the data were re-binned into *n *= 8, 10, 8 and 9 bins for the TRAV, TRAJ, TRGV and TRGJ trimming distributions, respectively. As shown in [28], since the parameters (*p*, *λ*) were initially estimated using maximum likelihood, the degree of freedom lies somewhere between *n *- 1 - *r *and *n *- 1, where *r *is the number of parameters estimated using maximum likelihood. We have thus that *r *= 2.

We found χ^2 ^= 7.97, 11.93, 7.27 and 31.62 for the TRAV, TRAJ, TRGV and TRGJ trimming distributions, respectively. For TRAV, TRAJ and TRGV, we find that at all standard values of statistical significance (*p *= 0.05, 0.01, 0.005), the null hypothesis is not rejected, and thus it is plausible that the empirical results follow a Bernoulli-Poisson-type law. However, for TRGJ, the null hypothesis is rejected at all of the same values of statistical significance. Thus, as it stands, the Bernoulli-Poisson law hypothesis would seem unlikely for the TRGJ trimming process.

## Conclusion

Exploiting standardized "output" datasets of IMGT/V-QUEST+JCTA, we have shown how to recover, under several hypotheses, a representation of the probability distributions of the "true" (or "bias-corrected") TRAV, TRAJ, TRGV and TRGJ trimming processes.

We proceeded by constructing a simple first-order model, known as the (4/3, 1/3) rule, followed by a second-order model [see Additional file 1] which had more general hypotheses. It it clear that the first-order model is a good approximation to the second-order model. We then showed that a kind of two-step Bernoulli-Poisson distribution could plausibly explain the transformed TRAV, TRAJ and TRGV trimming distributions.

We remark that for the TRA and TRG data available to us, the first-order model is "close" to the original IMGT/V-QUEST output data. This is partially due to the relatively smoothly varying data distributions being only slightly modified by performing the operation 4/3 ℙ {*F *= *k*} - 1/3 ℙ {*F *= *k *+ 1} (this would not necessarily be true for more irregular probability distributions). An implication of this, for biologists, is that when hypothesis testing on TRA and TRG data sets, as long as the data is relatively smoothly varying from one value to the next, there should be no problem using the IMGT/V-QUEST+JCTA output data, without transformation. Indeed, for our 4 data sets, the same hypothesis tests gave the same statistical result both on the IMGT/V-QUEST+JCTA output data as well as the first-order transformed data.

The statistical analysis of TR and IG junction sequences is a very young field due to the need of having large, clean datasets, unthought-of until recently. Since processes such as the trimming process examined in this article are very little understood from a physical point of view (i.e., what is the exact series of events? By which enzyme is trimming performed? How is exonuclease activity controlled [29]?), we see this work as opening a window to making hypotheses about the very nature of these physical processes and eventually improve our understanding of the complex molecular mechanisms of V-(D)-J recombination [30-33]. IMGT^® ^standardized criteria will eventually enable dealing with datasets numbering in the thousands or millions, impossible to deal with by hand. Under this framework of much larger datasets, we hope the present work will inspire improved models that eventually allow a series of specific, testable hypotheses to be made.

## Methods

### Datasets

T cell receptor (TR) genes were chosen for their absence of somatic hypermutation (in contrast to the IG) [9,10]. Among TR, the TRA and TRG rearrangements were selected because these loci have only two types of rearranging genes, V and J, in contrast to the TRB and TRD rearrangements which also have D genes [9]. The TRA dataset consisted of 212 human rearranged TRAV-TRAJ junction sequences, selected after alignment and analysis by the integrated IMGT/V-QUEST+JCTA software [23-25] and for which the output was agreed upon by experts (any sequence with potential but not yet confirmed allelic polymorphisms or with some unusual characteristics in the 3'V-REGION or 5'J-REGION was not included in the dataset). This same dataset was used in [12] to perform some preliminary statistical analyses.

An identical methodology was used to collate a dataset of 220 human rearranged TRGV-TRGJ junction sequences. Figures 2 and 3 show the IMGT/V-QUEST+JCTA output for the 'number of trimmed V (and J) nucleotides' for TRA and TRG, respectively.

### Junction analysis

The methodology for the detailed analysis of the junction is described in [25]. Briefly, IMGT/JunctionAnalysis [25] uses the 3'V-REGION of the 'best' aligned germline V gene and allele identified by IMGT/V-QUEST [23,24] to analyse the junction and delimit the 3' end of the 3'V-REGION in the analysed sequence (checking as far as possible in the 3' direction until encountering a nucleotide that is different from the germline 3'V-REGION, as by default no mutation is allowed for TR). Then, IMGT/JunctionAnalysis uses the 5'J-REGION of the 'best' aligned germline J gene and allele identified by IMGT/V-QUEST to delimit the 5' end of the 5'J-REGION in the analysed sequence (checking as far as possible in the 5' direction until encountering a nucleotide that is different from the germline 5'J-REGION, as by default no mutation is allowed for TR). The remaining nucleotides between the post-trimming 3'V-REGION and post-trimming 5'J-REGION nucleotides are denoted the N region (or if no trimming has occurred, short nucleotide sequences known as the P3'V-REGION or P5'J-REGION may be present [34,35]). The variables used for statistical analyses of TRA V-J junctions are described in [12]. The same variables were used for the TRG V-J junctions.

## Authors' contributions

KB developed the main mathematical and algorithmic arguments in the article. M-PL introduced the biological problem and ensured the validity of biological hypotheses. GB provided additional mathematical ideas and verified the theoretical results. All authors read and approved the final manuscript.

## Supplementary Material

Additional file 1 **Supplementary Data**. Statement and proof of first and second-order models, followed by a basic description of Bernoulli and Poisson distributions. Click here for file
