# Supplementary material for: Recovering probabilities for nucleotide trimming processes for T cell receptor TRA and TRG V-J junctions analyzed with IMGT tools
Source: BMC Bioinformatics. 2008 Oct 2;9:408. doi: 10.1186/1471-2105-9-408 (PMC2576266; doi:10.1186/1471-2105-9-408)
Supplement: Additional file 1 — Supplementary Data. Statement and proof of first and second-order models, followed by a basic description of Bernoulli and Poisson distributions. [file 1471-2105-9-408-S1.pdf]

1 Mathematics of the first and second-order models

We give here some details regarding the mathematical construction of the first and second-order models.

1.1 First-order model

Theorem 1. Suppose that N-REGION A, C, G and T nucleotides are uniformly distributed (i.e., equiprobable) and independent. Let $\mathcal{B}$ be the infinite vector

$$\mathcal{B} = (\,\mathbb{P}\{B = 0\},\, \mathbb{P}\{B = 1\},\, \mathbb{P}\{B = 2\}, \ldots)$$

and $\mathcal{F}$ the infinite vector

$$\mathcal{F} = (\,\mathbb{P}\{F = 0\},\, \mathbb{P}\{F = 1\},\, \mathbb{P}\{F = 2\}, \ldots)\,.$$

Then

$$\mathcal{B} \;=\; \frac{4}{3}\mathbf{A}^{-1}\mathcal{F},$$

where $\mathbf{A}$ is the infinite upper-diagonal matrix with, for $k \geq 1$, $\mathbf{A}_{1k} = (4/3)(1/4)^{k-i}$ and for $k \geq i > 1$, $\mathbf{A}_{ik} = (1/4)^{k-i}$.

Proof of Theorem 1. The fundamental remark is that if we see (i.e., the automatic tool gives) that for example 3 V nucleotides have been trimmed, then *at least* 3 nucleotides have been trimmed (in reality). To expand on this remark, we must first introduce the notation $\mathbb{P}\{Q \,|\, R\}$ to mean 'the probability that if the event R has occurred, then the event Q has occurred. The fundamental remark then can be written as: for non-negative integers $k < i$, $\mathbb{P}\{F = i \,|\, B = k\} = 0$. The law of total probability immediately gives that for any integer $i \geq 0$,

$$\sum_{k=i}^{\infty} \mathbb{P}\{F = i \,|\, B = k\}\mathbb{P}\{B = k\} \;=\; \mathbb{P}\{F = i\}.$$

Under the hypotheses of the theorem, it is easy to show that for $k \geq i$, if $i = 0$, then

$$\mathbb{P}\{F = 0 \,|\, B = k\} \;=\; \frac{1}{4^k}\;,$$

and if $i > 0$, then

$$\mathbb{P}\{F = i \,|\, B = k\} \;=\; \frac{1}{4^{k-i}} \times \frac{3}{4}.$$

In effect, each factor of 1/4 represents the probability that a trimmed V nucleotide is replaced by the exact same nucleotide. The factor of 3/4 means that, once the $k - i$ nucleotides are identically replaced, the next one *is not.* The probability of this, under our hypotheses, is 3/4 (thus when $i > 0$ we are essentially dealing with a geometric distribution). We have therefore

$$\sum_{k=i}^{\infty} \frac{3}{4} \frac{1}{4^{k-i}} \mathbb{P}\{B = k\} \;=\; \mathbb{P}\{F = i\},$$

except for $i = 0$, where the factor of 3/4 is omitted. To resolve these equations simultaneously for all $i \geq 0$, we form the (infinite) matrix equation

$$\frac{3}{4} \mathbf{A} \mathcal{B} \;=\; \mathcal{F},$$

where $\mathbf{A}$ is the matrix defined in the theorem statement. $\mathbf{A}$ is invertible and the explicit form of $\mathbf{A}^{-1}$ is given by $\mathbf{A}^{-1}_{11} = 3/4$, $\mathbf{A}^{-1}_{12} = -1/4$ and for all $q > 1$, $\mathbf{A}^{-1}_{qq} = 1$ and $\mathbf{A}^{-1}_{q\,q+1} = -1/4$. All other matrix entries are zero. The result follows. □

1.2 Second-order model

1.2.1 Introduction

One strategy to improve the model is to estimate the relative nucleotide frequencies for each region and attempt to prove a more general form of Theorem 1. One obvious problem is that there are many different genes (for example, 54 TRAV genes, not including alleles of each) and we have a dataset of only hundreds of junctions.

Thus, as we have no obvious way to estimate the proportion of cases each type of V or J gene is used in junction formation, we cannot easily estimate the global proportional use of A, C, G and T nucleotides in 3'V-REGIONs and 5'J-REGIONs. One possible strategy is to estimate the nucleotide frequencies *with what we have* but try not to use these exact numbers *explicitely* in the second-order model. Indeed, it turns out that if we suppose the frequencies of each nucleotide are fairly close to 1/4 (the range from 1/6 to 1/3) without specifying exactly each different frequency, we can get a more general result than Theorem 1.

First, to consider whether this was a reasonable hypothesis, we examined the set of TRAV, TRAJ, TRGV and TRGJ germline genes by collating each of their 15 3'-furthermost TRAV (and TRGV) and 5'-furthermost TRAJ (and TRGJ) nucleotides as a dataset. We then calculated nucleotide frequencies for each case. We found that the TRAV C nucleotide frequency was slightly below 1/6, G frequency slightly above 1/3 and the A and T frequencies between 1/6 and 1/3. For the TRAJ genes, all four frequencies were between 1/6 and 1/3. For the TRGV genes, the T frequency was slightly below 1/3, the G frequency close to 0.45 and the A and C frequencies between 1/6 and 1/3. For the TRGJ genes, the A and C frequencies were slightly above 1/3, the C frequency well below 1/6 and the G frequency between 1/6 and 1/3. Finally, we randomly sampled 50 N-REGIONS in the TRA and TRG cases. For the TRA, the A frequency was slightly below 1/6, the C frequency slightly above 1/3 and the G and T frequencies between 1/6 and 1/3. For the TRG, all four frequencies were between 1/6 and 1/3.

Since different genes are surely used with different frequency and not all 15 nucleotides are always trimmed, it is impossible to make a broad conclusion as to the validity of the new hypothesis. However, for the TRA case, the hypothesis seems reasonable. For the TRG case, there are very few different genes compared with the number of TRA genes, so the amount that each gene is used will be extremely critical as to the real-world frequencies. Nevertheless, the very high apparent G frequency for the TRGV genes and low C frequency for the TRGJ genes might encourage us to improve these mathematical models in the future if the frequencies are confirmed to be well out of the 1/3 to 1/6 range.

1.2.2 Mathematical modelling

To begin, we must introduce some notation. Let V_i, N_i and J_i be the nucleotide frequencies in the V, N, and J-REGIONs respectively, for $i \in$\{A, C, G, T\}. Also, let P_{VQ} be the probability that a randomly chosen V nucleotide is the same as a randomly chosen Q (Q = N or J) nucleotide. With this notation, we see that

$$P_{VQ} \;=\; V_A Q_A + V_C Q_C + V_G Q_G + V_T Q_T.$$

Since it will be useful to bound this quantity independently of whether Q = N or J, we have the following lemma.

Lemma 1. Suppose that $0 \le y \le 1/12$ and that for each of V_i, N_i and J_i we have

$$\frac{1}{4} - y \;\le\; V_i, N_i, J_i \;\le\; \frac{1}{4} + y.$$

Then for Q = N or J we have that

$$\frac{1}{4} - 4y^2 \;\le\; P_{VQ} \;\le\; \frac{1}{4} + 4y^2.$$

Proof of Lemma 1. We prove only $P_{VQ} \le 1/4 + 4y^2$. The other statement follows an analogous proof. Denoting $V = (V_A, V_C, V_G, V_T)$ and $Q = (Q_A, Q_C, Q_G, Q_T)$, without loss of generality suppose that

$$V \;=\; Q \;=\; \left(\frac{1}{4} - y,\; \frac{1}{4} - y,\; \frac{1}{4} + y,\; \frac{1}{4} + y\right).$$

Then it is easy to calculate $P_{VQ} = 1/4 + 4y^2$. We want to show that this maximum can't be beaten. Necessarily, if we want to calculate P_{VQ} for a different set of V-REGION and Q-REGION probabilities, we are obliged to increase the values of V_A, V_C, Q_A and Q_C by some non-negative ϵ_1, ϵ_2, ϵ_5 and ϵ_6, respectively, and correspondingly decrease the values of V_G, V_T, Q_G and Q_T by some non-negative ϵ_3, ϵ_4, ϵ_7 and ϵ_8 respectively. It is easy to see that we have the constraints $\epsilon_i \le 2y$ for $i \in \{1, \ldots, 8\}$, $\epsilon_1 + \epsilon_2 = \epsilon_3 + \epsilon_4$ and $\epsilon_5 + \epsilon_6 = \epsilon_7 + \epsilon_8$. We can then write the perturbation of (V_A, V_C, V_G, V_T) as

$$V' \;=\; \left(\frac{1}{4} - y + \epsilon_1, \frac{1}{4} - y + \epsilon_2, \frac{1}{4} + y - \epsilon_3, \frac{1}{4} + y - \epsilon_4\right),$$

and the perturbation of (Q_A, Q_C, Q_G, Q_T) as

$$Q' \;=\; \left(\frac{1}{4} - y + \epsilon_5, \frac{1}{4} - y + \epsilon_6, \frac{1}{4} + y - \epsilon_7, \frac{1}{4} + y - \epsilon_8\right).$$

Upon calculating (and using the constraints on each ϵ_i), we find

$$V' \cdot Q' \;=\; V \cdot Q \;+\; \epsilon_1\epsilon_5 + \epsilon_2\epsilon_6 + \epsilon_3\epsilon_7 + \epsilon_4\epsilon_8 \;-\; 2y(\epsilon_3 + \epsilon_4 + \epsilon_7 + \epsilon_8).$$

Again using the constraints on each ϵ_i, we see that

$$\epsilon_1\epsilon_5 + \epsilon_2\epsilon_6 + \epsilon_3\epsilon_7 + \epsilon_4\epsilon_8 \;\le\; 2y(\epsilon_1 + \epsilon_2 + \epsilon_7 + \epsilon_8) \;=\; 2y(\epsilon_3 + \epsilon_4 + \epsilon_7 + \epsilon_8).$$

Thus we have that $V' \cdot Q' \le V \cdot Q$.

To prove the second inequality, it suffices to interchange the values of V_A and V_G, and the values of V_C and V_T, and show that the resulting value of $V \cdot Q$ is a global minimum for this constrained optimization problem. □

1.2.3 Differences with the first-order model

Whereas before we were able to deal with a matrix with simple geometric symmetry, here some of that symmetry is gone, meaning that matrix inversion is not so straightforward. The reason for this is that it is no longer true that for $k \geq i$, if $i = 0$, then

$$\mathbb{P}\{F = 0 \,|\, B = k\} \;=\; \frac{1}{4^k},$$

and if $i > 0$, then

$$\mathbb{P}\{F = i \,|\, B = k\} \;=\; \frac{1}{4^{k-i}} \times \frac{3}{4}.$$

To take an example, suppose we want to calculate $\mathbb{P}\{F = 2 \,|\, B = 4\}$, the probability that if 4 V nucleotides were trimmed, we 'see' only 2 in the tool output. For this to happen, the two nucleotides from the N-REGION/5'J-REGION closest to the trimmed 3'V-REGION would have to accidentally match the 2 5'−furthermost trimmed 3'V-REGION nucleotides, and the third closest nucleotide from the N-REGION/5'J-REGION would have to not match the 3rd 5'−furthermost trimmed 3'V-REGION nucleotide. Since the nucleotide frequencies in the 3'V-REGION, N-REGION and 5'J-REGION are not known exactly, the value of $\mathbb{P}\{F = 2 \,|\, B = 4\}$ becomes essentially unknown. Furthermore, the value of $\mathbb{P}\{F = 2 \,|\, B = 4\}$ will change depending on whether the N-REGION contained 0, 1, 2 or more nucleotides, making the calculation seemingly impossible as we would also need good information about the true length distribution of the N-REGION.

Thankfully, by just assuming that the nucleotide frequencies are bounded as in Lemma 1, it turns out that progress can be made.

1.2.4 A symbolic approach

Our ultimate goal is to develop a method which allows us to invert a matrix of bounded numbers, even though we don't know exactly what each number is. Therefore, this is not strictly the inverse of a matrix, since we cannot calculate the explicit numerical values of this inverse. However, it turns out that we can calculate a bounded inverted matrix, in the sense that the entries of this inverted matrix are bounded and the bounds can be calculated explicitely. Indeed, by showing these bounds are tight, we will be able to show that the second-order method is merely a small perturbation of the first-order method.

In order to develop this method, we need to simplify notation even further. Let Z be given the same meaning as P_{VQ}. i.e., Z is any $P_{VQ}-$type object satisfying $1/4 - 4y^2 \leq Z \leq 1/4 + 4y^2$. We will denote I the compact $I = [1/4 - 4y^2, 1/4 + 4y^2]$. Furthermore, for any mathematical statement that includes Z's, the only algebraic operations that can be performed on it are operations which preserve the meaning of each Z as a bounded, yet unknown object.

Essentially we will need algebraic operations that loosen inequalities or keep them the same. We will use the notation $M \leftarrow N$ to mean that M can legally be replaced by N. This means in effect that the bounds on N loosen (or keep the same) the bounds on M.

To continue, let us denote by $f(Z)$ (or $g(Z)$) some strictly positive function of any number ℓ of bounded variables $Z_1, \ldots, Z_\ell$. To give an explicit example, we could have

$$f(Z) = \frac{(Z_1)^3(1 - Z_2)^4}{(1 - Z_3)^6}\,.$$

Whilst it may seem strange at first, it is actually simpler to write this example as

$$f(Z) = \frac{(Z)^3(1 - Z)^4}{(1 - Z)^6}\,, \tag{1.1}$$

since in the following development, mathematical statements will include varying number of different Z variables, making the notation more complicated than it needs to be. The important thing to remember is thus that if $f(Z)$ is written as in (1.1), each Z is not necessarily the same number, though each is subject to the same bounds. In particular, if we wanted to find how big we could make $f(Z)$, we would have to calculate

$$\max f(Z) = \frac{\max_{Z\in I}\{Z^3\}\,\max_{Z\in I}\{(1 - Z)^4\}}{\min_{Z\in I}\{(1 - Z)^6\}}.$$

That is, we would have to treat each Z as being potentially different from the other Z's in order to calculate the maximum.

Under this initially strange notation, we must discover how to treat objects such as $f(Z) \times g(Z)$ or even $f(Z)/f(Z)$, which is no longer necessarily equal to 1. In fact, we need to compile a list of operations which can be performed that retain or loosen the bounds on functions, products of functions, quotients of functions, etc. The following list does just this, along with a practical example for each operation, and weighs heavily on the fact that $f(Z)$ and $g(Z)$ are strictly positive functions.

- For $a, b > 0$ or $a, b < 0$, $af(Z) + bf(Z) \leftarrow (a+b)f(Z)$.
Example:
$$2^3 \frac{Z}{1-Z} + 2^5 \frac{Z}{1-Z} \quad \leftarrow \quad (2^3 + 2^5)\frac{Z}{1-Z}.$$
In this simple case, the bounds of the right hand side are actually the same as those of the left hand side. It is therefore a legal operation.

- for m and n positive integers, $f(Z)^m \times f(Z)^n \leftarrow f(Z)^{m+n}$.
Example:
$$Z^3 \times Z^5 \quad \leftarrow \quad Z^8\,.$$
In this case, the bounds of the right hand side are also the same as the left hand side since $f(Z) = Z^3$ and $g(Z) = Z^5$ are strictly positive bounded functions of the same bounded variable Z.

- $1 \leftarrow f(Z)/f(Z)$. The converse is not true, in general.
Example:
$$1 \quad \leftarrow \quad \frac{Z(1-Z)}{Z(1-Z)}.$$
In effect, on choosing suitable (and different) values for Z in the numerator and denominator, the right hand side can be made either greater than or less than 1. Thus the bounds are loosened.

- $0 \leftarrow f(Z) - f(Z)$. The converse is not true, in general.
Example:
$$0 \quad \leftarrow \quad \frac{Z(1-Z)^2}{(1-Z)^3} - \frac{Z(1-Z)^2}{(1-Z)^3}.$$
In effect, the right hand side can be made positive or negative by choosing suitable (different) values of Z. Thus the bounds are loosened.

- For any integers $a, b, c, d, e, f \;\geq\; 0$,
$$\frac{Z^a(1-Z)^b}{(1-Z)^c} \times \frac{Z^d(1-Z)^e}{(1-Z)^f} \quad \leftarrow \quad \frac{Z^{a+d}(1-Z)^{b+e}}{(1-Z)^{c+f}}. \tag{1.2}$$
Example: Any choice of a, b, c, d, e, f gives an example.

- $(f(Z) - f(Z))\, g(Z) \;\leftarrow\; f(Z)g(Z) - f(Z)g(Z)$.
Example:
$$\left\{\frac{Z(1-Z)}{1-Z} - \frac{Z(1-Z)}{1-Z}\right\} \frac{Z^2}{(1-Z)^2}$$

$$\leftarrow \quad \frac{Z(1-Z)}{1-Z} \times \frac{Z^2}{(1-Z)^2} \quad - \quad \frac{Z(1-Z)}{1-Z} \times \frac{Z^2}{(1-Z)^2}$$

Using (1.2), this could then be simplified further.

With this established methodology, we can symbolically calculate a matrix as before, keeping the entries as unknown bounded functions of Z, and try to invert it using legal operations. To start, for $k \geq i$, if $i = 0$, we have

$$\mathbb{P}\{F = i \,|\, B = k\} \;\leftarrow\; Z^k,$$

and if $i > 0$, then

$$\mathbb{P}\{F = i \,|\, B = k\} \;\leftarrow\; Z^{k-i}(1-Z).$$

We will store all of this information in a matrix $\mathbf{A}$, excluding the first row and column (for $i = 0$), as this special case is easier to treat separately. By denoting for $i > 0$ and $j \geq 1$, $y_j \;=\; \mathbb{P}\{F = i \,|\, B = i+j-1\} \;=\; Z^{j-1}(1-Z)$, our system of equations as before can be stored as

$$\mathbf{A} = \begin{pmatrix} y_1 & y_2 & y_3 & y_4 & y_5 & \cdots \\ 0 & y_1 & y_2 & y_3 & y_4 & \cdots \\ 0 & 0 & y_1 & y_2 & y_3 & \cdots \\ 0 & 0 & 0 & y_1 & y_2 & \cdots \\ 0 & 0 & 0 & 0 & y_1 & \cdots \\ \vdots & \vdots & \vdots & \vdots & \vdots & \ddots \end{pmatrix}.$$

Whilst we have lost some (geometric series) symmetry as compared with before, there is nevertheless enough symmetry to see that $\mathbf{A}^{-1}$ will have the following form:

$$\mathbf{A}^{-1} = \begin{pmatrix} x_1 & x_2 & x_3 & x_4 & x_5 & \cdots \\ 0 & x_1 & x_2 & x_3 & x_4 & \cdots \\ 0 & 0 & x_1 & x_2 & x_3 & \cdots \\ 0 & 0 & 0 & x_1 & x_2 & \cdots \\ 0 & 0 & 0 & 0 & x_1 & \cdots \\ \vdots & \vdots & \vdots & \vdots & \vdots & \ddots \end{pmatrix}.$$

We note that since the matrix $\mathbf{A}$ is upper diagonal, we can safely treat the special case ($i = 0$) later. We see immediately that $x_1 \leftarrow 1/y_1$ and for $i \geq 2$,

$$x_i \;\leftarrow\; -\sum_{m=1}^{i-1} \frac{y_{i-m+1}}{y_1}\, x_m, \tag{1.3}$$

which could easily be solved recursively, if the y_i didn't happen to be functions of unknown Z's. Instead, we solve them symbolically, by performing legal operations on the bounded unknown functions of Z, as shown in the following lemma.

Lemma 2. The system of equations (1.3) can be solved symbolically, yielding as solutions

$$x_1 \leftarrow \frac{1}{1-Z} ,$$

$$x_2 \leftarrow -\frac{Z(1-Z)}{(1-Z)^2} ,$$

and for $j \geq 3$,

$$x_j \leftarrow 2^{j-3} \left[\frac{Z^{j-1}(1-Z)^{j-1}}{(1-Z)^j} \right] - 2^{j-3} \left[\frac{Z^{j-1}(1-Z)^{j-1}}{(1-Z)^j} \right] .$$

Proof of Lemma 2. By induction. To show it is true for x_3 is straightforward, by directly using the legal operations given in the previous list. We suppose it is true for $x_3, \ldots, x_k$ and show it is true for x_{k+1}, using all of the legal transformations. We have that

$$x_{k+1} \leftarrow -\sum_{m=1}^{k} \frac{y_{k-m+2}}{y_1} x_m .$$

Expanding, we find

$$\begin{aligned} x_{k+1} \leftarrow & -\frac{Z^k(1-Z)}{(1-Z)^2} + \frac{Z^k(1-Z)^2}{(1-Z)^3} \\ & - \frac{Z^{k-2}(1-Z)}{(1-Z)} \times \left[2^0 \frac{Z^2(1-Z)^2}{(1-Z)^3} - 2^0 \frac{Z^2(1-Z)^2}{(1-Z)^3} \right] - \\ & \ldots - \frac{Z(1-Z)}{(1-Z)} \times \left[2^{k-3} \frac{Z^{k-1}(1-Z)^{k-1}}{(1-Z)^k} - 2^{k-3} \frac{Z^{k-1}(1-Z)^k}{(1-Z)^k} \right] . \end{aligned}$$

By multiplying through so that all denominators become $(1-Z)^{k+1}$ (this is a combination of legal operations), and then adding like terms (another legal operation), we obtain

$$\begin{aligned} x_{k+1} \leftarrow & \left\{ 1 + 2^0 + \ldots + 2^{k-3} \right\} \frac{Z^k(1-Z)^k}{(1-Z)^{k+1}} \\ & + \left\{ -1 - 2^0 - \ldots - 2^{k-3} \right\} \frac{Z^k(1-Z)^k}{(1-Z)^{k+1}} \end{aligned}$$

$$\leftarrow \; 2^{k-2}\left[\frac{Z^k(1-Z)^k}{(1-Z)^{k+1}}\right] \; - \; 2^{k-2}\left[\frac{Z^k(1-Z)^k}{(1-Z)^{k+1}}\right]. \quad \square$$

Remark: Upon taking $Z = 1/4$, we fall back onto the first-order approximation, giving $x_1 = 4/3$, $x_2 = -1/3$ and $x_i = 0$ for $i \geq 3$.

It is now straightforward to prove the following theorem.

Theorem 2. Suppose that Z is any $P_{VQ}-$type object satisfying

$$1/4 - 4y^2 \;\leq\; Z \;\leq\; 1/4 + 4y^2.$$

Then for $k \geq 1$ we have

$$\mathbb{P}\{B = k\} \;=\; \left\{\frac{4}{3} + C_1\right\}\mathbb{P}\{F = k\} \;-\; \left\{\frac{1}{3} + C_2\right\}\mathbb{P}\{F = k+1\} \;+\; \epsilon_k \;,$$

where $|\,C_1| \;<\; 0.052$, $|\,C_2| \;<\; 0.081$ and

$$|\,\epsilon_k| \;<\; \frac{1}{2}\sum_{j=3}^{\infty}\left(\frac{3}{4}\right)^j \mathbb{P}\{F = k+j-1\}.$$

Proof of Theorem 2 The proof is simple. We take $y = \pm 1/12$, it's extremums. Then, bounding x_1 gives C_1, x_2 gives C_2 and $x_3, x_4, \ldots$ gives ϵ_k, respectively. $\square$

Remark: The bound on ϵ_k is not chosen to be tight but merely to show that it is a converging geometric sum. Indeed, if we write instead

$$|\,\epsilon_k| \;<\; \sum_{j=3}^{\infty}\mu_j \;\mathbb{P}\{F = k+j-1\},$$

by not oversimplifying the inequalities used to bound $x_3, x_4, \ldots$, it is simple to show that $|\,\mu_3| \;<\; 0.070$, $|\,\mu_4| \;<\; 0.052$, $|\,\mu_5| \;<\; 0.035$, $|\,\mu_6| \;<\; 0.023$, $|\,\mu_7| < 0.015$ and $|\,\mu_8| < 0.009$. Whilst the exact bound on ϵ_k will depend on the law f_F of F, it is nevertheless clear that for any reasonable f_F, the value of ϵ_k is going to have an insignificant effect on the value of $\mathbb{P}\{B = k\}$.

Remark: We see thus that the second-order model can be represented as a small perturbation of the first-order model.

To complete this section, we must deal with the special case of when $i = 0$, that is, the set of probabilities for when zero trimming is 'seen' (by the automatic tool). Recall that for $k \geq i$, if $i = 0$, we have

$$\mathbb{P}\{F = i \,|\, B = k\} \;=\; Z^k.$$

We take the matrix $\mathbf{A}$ and give it back the necessary extra row and column, calling it now $\mathbf{A}_2$:

$$\mathbf{A}_2 = \begin{pmatrix} y_{00} & y_{01} & y_{02} & y_{03} & y_{04} & y_{05} & \cdots \\ 0 & y_1 & y_2 & y_3 & y_4 & y_5 & \cdots \\ 0 & 0 & y_1 & y_2 & y_3 & y_4 & \cdots \\ 0 & 0 & 0 & y_1 & y_2 & y_3 & \cdots \\ 0 & 0 & 0 & 0 & y_1 & y_2 & \cdots \\ 0 & 0 & 0 & 0 & 0 & y_1 & \cdots \\ \vdots & \vdots & \vdots & \vdots & \vdots & \vdots & \ddots \end{pmatrix}.$$

To clarify notation, we have for $k \geq 0$, $y_{0k} \;=\; \mathbb{P}\{F = 0 \,|\, B = k\} \;=\; Z^k$. Correspondingly, $\mathbf{A}_2$ will have as inverse

$$\mathbf{A}_2^{-1} = \begin{pmatrix} x_{00} & x_{01} & x_{02} & x_{03} & x_{04} & x_{05} & \cdots \\ 0 & x_1 & x_2 & x_3 & x_4 & x_5 & \cdots \\ 0 & 0 & x_1 & x_2 & x_3 & x_4 & \cdots \\ 0 & 0 & 0 & x_1 & x_2 & x_3 & \cdots \\ 0 & 0 & 0 & 0 & x_1 & x_2 & \cdots \\ 0 & 0 & 0 & 0 & 0 & x_1 & \cdots \\ \vdots & \vdots & \vdots & \vdots & \vdots & \vdots & \ddots \end{pmatrix}.$$

We see immediately that $x_{00} = 1$ and for $j \geq 1$,

$$x_{0j} \;=\; -\sum_{m=1}^{j} y_{0\,j-m+1}\, x_m, \tag{1.4}$$

which can be solved symbolically as before since all of the x_m and y_{0k} are already known. The following corollary to Theorem 2 gives the solution in a form analagous to the result in Lemma 2.

Corollary 1. The system of equations (1.4) can be solved symbolically, yielding as solutions $x_{00} = 1$, $x_{01} = -Z/(1-Z)$ and for $j \geq 2$,

$$x_{0j} \;=\; 2^{j-2}\left[\frac{Z^j(1-Z)^{j-1}}{(1-Z)^j}\right] \;-\; 2^{j-2}\left[\frac{Z^j(1-Z)^{j-1}}{(1-Z)^j}\right].$$

Furthermore, we have

$$\mathbb{P}\{B=0\} \;=\; \mathbb{P}\{F=0\} \;-\; \left\{\frac{1}{3}+C_3\right\}\mathbb{P}\{F=1\} \;+\; \epsilon_0\ ,$$

where $|\,C_3| \;<\; 0.052$ and

$$|\,\epsilon_0| \;<\; \frac{1}{2}\sum_{j=3}^{\infty}\left(\frac{3}{4}\right)^j\,\mathbb{P}\{F=j-1\}.$$

Proof of Corollary 1 The result for x_{0j} can be proven by induction exactly as in Lemma 2. The results for C_3 and ϵ_0 come from bounding the resulting x_{0j}'s as in Theorem 2. $\square$

Remark: As in Theorem 2, the bound ϵ_0 is not chosen to be tight but merely to show that it is a converging geometric sum. Indeed, if we write instead

$$|\,\epsilon_0| \;<\; \sum_{j=3}^{\infty}\mu_j\ \mathbb{P}\{F=j-1\},$$

by not oversimplifying the inequalities used to bound x_{02}, $x_{03},\ldots$, it is simple to show that $|\,\mu_3| \;<\; 0.057$, $|\,\mu_4| \;<\; 0.045$, $|\,\mu_5| \;<\; 0.032$, $|\,\mu_6| \;<\; 0.021$, $|\,\mu_7| \;<\; 0.013$ and $|\,\mu_8| \;<\; 0.009$. Again, whilst the exact bound on ϵ_0 will depend on the law f_F of F, it is nevertheless clear that for any reasonable f_F, the value of ϵ_0 is going to have an insignificant effect on the value of $\mathbb{P}\{B=0\}$.

2 A basic description of Bernoulli and Poisson distributions

2.1 Bernoulli distributions

A Bernoulli distribution is the result of a random process with only two fixed-probability outcomes. A simple example is the toss of a coin: if the coin is fair, we toss heads with probability 1/2 and tails with probability 1/2. Another example is a farmer with cows that are either sick or healthy. If ten percent of his cows are sick and he randomly chooses one cow from the herd, the probability it is a sick one is 1/10 and the probability it is healthy is 9/10.

We say here that cow sickness follows a Bernoulli distribution with parameter $p = 1/10$. Tossing a fair coin gives a Bernoulli distribution with parameter $p = 1/2$.

The possible relation to trimming processes came about from the observation that there were significant quantities of data at zero. A potential conclusion was that, sometimes, the trimming did not start (that is, the conditions in the cell did not allow trimming to occur). Thus the process had two outcomes: either trimming was allowed to proceed, or it was not. Naturally, this suggested modelling using a Bernoulli process. We estimated the parameter p and used it later in the hypothesis testing to see if overall, we could be dealing with a Bernoulli distribution followed by a Poisson process.

2.2 Poisson distribution

A Poisson distribution is the result of counting the number of independent events (photons hitting a photodetector, particles emmitted via radioactive decay, etc.) that occur in a fixed interval of time, repeated many times. Independence, mathematically speaking, means that the fact that one event occurs (or does not occur) has no influence on whether another event occurs or not. Another simple example of independence would be that the moment (day, time) one person drives their car to a gas station has no effect on the moment another person, unknown to them, drives their car to the same gas station.

If we fix the gas station, and count the number of cars that arrive each ten minutes, and this for many blocks of ten minutes, the resulting histogram "count" (number of periods which had 0, 1, 2, 3,. . . cars) gives what is called

a Poisson distribution. Note that this example is instructive rather than perfect, as it could be argued that at certain times of the day or days of the week naturally more people fill up their cars, but it illustrates the general idea.

The possible relation to trimming processes is the following: we saw often that the real-life data (after trying to remove tool biases) that was available to us took histogram shapes that suggested Poisson-like processes. The present article suggests that biologists looking to characterize the real physical processes involved in trimming need to present theories consistent with the probability distributions found in this article.
